# Association of interferon gamma inducible protein-10, monocyte chemoattractant protein-1, macrophage inflammatory protein-1 alpha, interleukin-6, and rs12252 single nucleotide polymorphism of interferon-induced transmembrane protein-3 gene with the severity of COVID-19 infection

**DOI:** 10.1186/s43162-022-00141-9

**Published:** 2022-07-08

**Authors:** Shafia Mulla, Md.Maruf Ahmed Molla, S. M. Ali Ahmed, A. K. M. Akhtaruzzaman, Ahmed Abu Saleh, Shaheda Anwar

**Affiliations:** 1 Department of Microbiology &Immunology, Bangabandhu Sheikh Mujib Medical University, Dhaka, 1217 Bangladesh; 2 Department of Virology, National Institute of Laboratory Medicine and Referral Center, Dhaka, 1207 Bangladesh; 3 Biomedical Research Program, State University of New York Upstate Medical University, Syracuse, 13208 USA; 4 Department of Anaesthesia, Analgesia and Intensive Care Medicine, Bangabandhu Sheikh Mujib Medical University, Dhaka, 1217 Bangladesh

**Keywords:** IP-10, MCP-1, MIP-1α, IL-6, IFITM3, COVID-19, SNP

## Abstract

**Background:**

Evaluating the serum levels of IP-10, MCP-1, MIP-1α, and IL-6 and genotyping of rs12252 SNP of *IFITM3* gene among different categories of COVID-19 patients might aid in understanding the pathogenesis of COVID-19 and contribute to developing disease-specific biomarkers and therapeutic strategies.

**Methods:**

This is a cross-sectional study involving a total of 84 COVID-19 patients confirmed by positive RT-PCR and 28 healthy controls. COVID-19 patients were recruited from the intensive care unit (ICU) and COVID unit of Bangabandhu Sheikh Mujib Medical University, Shahbag, Dhaka. COVID-19 patients were categorized into moderate, severe, and critically ill groups according to the World Health Organization classification. The serum IP-10, MCP-1, and MIP-1α levels were measured by cytometric bead array assay by flow cytometry, and serum IL-6 level was detected by the chemiluminescence method. rs12252 SNP of the *IFITM3* gene was detected by polymerase chain reaction-restriction fragment length polymorphism (PCR RFLP).

**Results:**

The serum IP-10, MCP-1, MIP-1α, and IL-6 levels among critically ill COVID-19 patients were significantly higher than that in patients with moderate disease and healthy controls (*p* < 0.001). Genotype distribution for rs12252 (42 T/C) SNP of the *IFITM3* gene between the different groups of COVID-19 patients and healthy controls showed that CC genotype was statistically associated with disease severity (*p* < 0.001).

**Conclusions:**

IP-10 and MCP-1, MIP-α, IL-6, and CC genotype of rs12252 (42 T/C) SNP of *IFITM3* gene are associated with COVID-19 severity.

## Introduction

Severe acute respiratory syndrome coronavirus 2 (SARS-CoV-2) is an enveloped, positive-sense, single-stranded RNA virus that is closely related to original SARS-CoV and Middle East respiratory syndrome (MERS) coronaviruses [1]. SARS-CoV-2 enters human cells with the help of angiotensin-converting enzyme 2 (ACE-2) located on the cell surface of different organs such as the heart, lung, kidney, testis, and digestive tract [2]. As a consequence of SARS-CoV-2 infection, several inflammatory factors are released forming a cytokine storm, which leads to a systemic inflammatory response syndrome (SIRS) [2].

Previous studies found out that patients with COVID-19 pneumonia have higher plasma levels of interleukin-1 beta (IL-1β), interleukin 2R (IL-2R), interleukin-6 (IL-6), interleukin-8 (IL-8), interleukin-10 (IL-10), interferon gamma (INF-γ), granulocyte colony-stimulating factor (G-CSF), interferon gamma-induced protein 10 (IP-10), monocyte chemoattarcttant protein-1 (MCP-1), macrophage inflammatory protein-1 alpha (MIP-1α), and tumor necrosis factor alpha (TNF-α) [3, 4]. In addition, it was revealed that ICU patients have higher concentrations of G-CSF, IP-10, MCP-1, MIP-1α, and TNF-α than non-ICU patients [5].

IP-10 prevents endothelial healing by blocking calpains-mediated angiogenesis [6]. Expression level of IP-10 was found to be highest among critically ill patients, followed by patients with severe and moderate COVID-19 disease. MCP-1 is a chemokine that acts by binding with chemokine receptor 2 (CCR2), which in turn attracts monocytes and basophils [7]. Macrophage inflammatory protein-1 alpha (MIP-1α) is a monocyte cytokine with inflammatory and chemotactic properties. It can interact with chemokine receptors CCR1, CCR4, and CCR5 [8]. SARS-CoV-2 infection of lung tissue induces the release of pro-inflammatory cytokines and chemokines including MCP-1 and MIP-1α [9]. Serum levels of MCP-1 and MIP-1α were found to be correlated with COVID-19 disease severity, and these cytokines levels are significantly higher in critically ill patients [10].

In SARS-CoV-2 infection, IL-6 is an important inflammatory interleukin that could possibly be antagonized by available drugs [11]. Only few cells such as macrophages, neutrophils, CD4 + T-cells, podocytes, and hepatocytes express IL-6 R on their surface. The serum levels of IL-6 are usually higher in critically ill COVID-19 patients than in severe and moderate patients [12]. IL-6 levels were found to be strongly correlated with mortality of COVID-19 patients [13].

Interferon-induced transmembrane protein-3 (IFITM3) protein also causes alteration of lipid/cholesterol content and curvature of endosomal membrane that in turn increases pH of endosomal contents, which favors killing of the virus [14]. Three single nucleotide polymorphisms (SNPs) of *IFITM3* gene rs34481144, rs6598045, and rs12252 (c. 42 T/C) were found to be associated in COVID-19 infection [15]. rs34481144 and rs6598045 SNPs are located in the 5′ untranslated region (5′-UTR) and proximal promoter, respectively, whereas rs12252 (c. 42 T/C) SNP is located in the coding region (Exon-1) and can significantly alter the expression level of the *IFITM3* protein. rs12252 SNP plays a significant role in the antiviral activity of these protein [16]. Frequency of the *IFITM3* rs12252 SNP risk variant (C) is found in about 52% of East Asian and 15% of South Asian population. In a previous study, Zhang et al. observed that a significantly higher frequency of rs12252 C allele carriers are patients with severe COVID-19 disease compared to mild patients. Their study revealed that among hospitalized patients, 35% are homozygous CC, 46.25% heterozygous TC, and 18.75% TT genotypes [17]. 

In the Bangladeshi population, there is no data regarding the association of *IFITM3* gene polymorphism and disease progression in COVID-19 patients. It will help in better understanding of disease pathogenesis, immune responses, susceptibility, and responsiveness of treatment and identify genetically high-risk persons in the Bangladeshi population. Besides, no study could be found that evaluated potential biomarkers of COVID-19 disease severity among Bangladeshi COVID-19 patients. While there are a significant number of studies illustrating the relationship between IP-10, MCP-1, MIP-1α, and IL-6, and COVID-19 disease severity in other countries, evaluating these cytokines in a population, where the COVID-19 morbidity and mortality rates have been very low, could add a different perspective to evolving understanding of COVID-19 disease pathogenesis and treatment.

## Materials and methods

This cro**ss**-sectional analytic study was conducted from March 2021 to January 2022 at the Department of Microbiology & Immunology; Department of Anesthesia, Analgesia and Intensive Care Medicine; and COVID-19 Unit in Bangabandhu Sheikh Mujib Medical University (BSMMU), Shahbag, Dhaka.

### Study population

#### Patient group

A total of 84 COVID-19 patients confirmed by positive RT-PCR for SARS-CoV-2 from nasopharyngeal or oropharyngeal swabs were enrolled in this study. Patients were recruited from the intensive care unit (ICU) and COVID-19 unit of BSMMU, Shahbag, Dhaka, and categorized into different severity groups according to the National Guideline on Clinical Management of COVID-19, version 9.0, published on 6 May 2021, Bangladesh.

#### Inclusion criteria

Moderate, severe, and critically ill COVID-19 patients of all age groups and genders confirmed by positive reverse transcriptase polymerase chain reaction (RT-PCR) test were included in this study.

#### Exclusion criteria

Patients who were taking immunosuppressive drugs and chemotherapy; patients having other known concomitant infections, such as tuberculosis, hepatitis, and HIV; and patients having known connective tissue disorders such as systemic lupus erythematosus (SLE) and rheumatoid arthritis were excluded from this study.

#### Control group

A total of 28 healthy subjects were included from the general population of the same geographic area. Healthy subjects were selected from the persons who attended fever clinic, BSMMU for COVID-19 screening test for travel purposes, and who were RT-PCR negative for COVID-19 and did not have any contact history and clinical symptoms of COVID-19 for the last 2 weeks.

#### Cytokine measurement

Serum was obtained by centrifugation of 3 ml whole blood sample at 4000 rpm for 5 min and stored at − 80 °C up to the study period. Serum IP-10 (catalog no. 558280), MCP-1 (catalog no. 558287), and MIP-1α (catalog no. 558325) levels were measured by using the BD Cytometric Bead Array Flex Kit, and IL-6 level was determined by chemiluminescence immunoassay (Beckman Coulter, USA) according to the manufacturer’s instruction.

### Molecular analysis

**Fig. 1:**
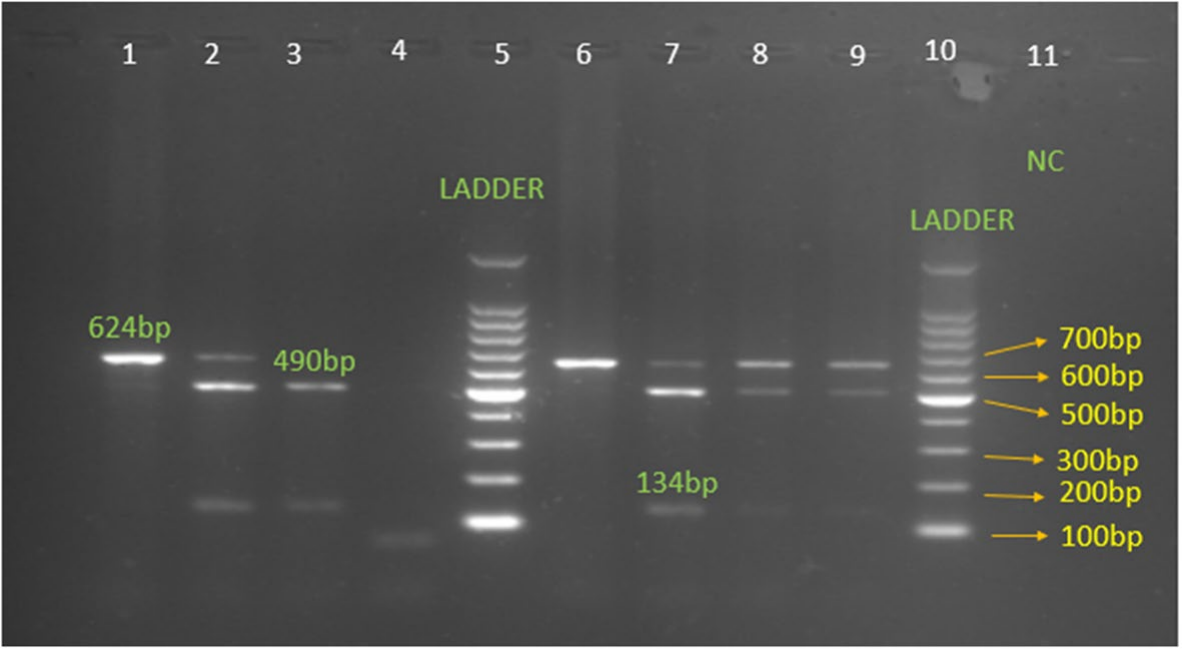
PCR–RFLP analysis of rs12252 (42 T/C) polymorphism of *IFITM3* gene by digestion with *Mscl* enzyme and separated by agarose gel electrophoresis. Lanes 1 and 6 show the CC genotype (624 bp), lane 3 shows the TT genotype (490 bp and 134 bp), and lanes 2 and 7 show the TC genotype (624 bp, 490 bp, 134 bp)

The SNP rs12252 was analyzed using PCR–RFLP. Blood samples were collected from all subjects in an EDTA tube, and genomic DNA was extracted from peripheral blood leukocytes by the standard spin column method (Genomic DNA extraction spin kit v2, Turkey). The entire coding region of the *IFITM3* gene was amplified by PCR using one pair of primer, ACTGTTGAGAAACCGAAACTACTGG (F) and CTATAGGGAGAACTGCTCTGGCT (R). Amplification was carried out in an ASTEC thermal cycler (Gene Atlas G, Japan) at 95 °C for 5 min, followed by 40 cycles of denaturation at 98 °C for 10 s, annealing at 68 °C for 30 s, and extension at 72 °C for 150 s. The final extension was completed at 72 °C for 7 min. *Mscl* enzyme (New England Biolabs, Ipswich, MA, USA) was used to digest the PCR product in the presence of the T allele (wild). Fragments with a length of 624, 490, and 134 base pairs were observed. The genotypes (TT, TC, CC) were identified by using agarose (2%) gel electrophoresis and stained with ethidium bromide (1%) (Fig. 1).

### Statistical analysis

All statistical analyses were performed using the Statistical Package for Social Science (SPSS) program (version-26). Continuous data were expressed as mean (± SD) and categorical data as frequency and percentage. Comparisons of categorical data were performed using the chi-square test or Fisher exact test. As the IP-10, MCP-1, MIP-1α, and IL-6 levels were skewed, the Mann–Whitney *U* test or Kruskal–Wallis analysis was done to compare the continuous data. The strength of association was assessed using odds ratio (OR) and 95% confidence interval (95% CI). *p-*value less than 0.05 was considered to be statistically significant.

### Ethical consideration

Ethical clearance was taken from the Institutional Review Board (IRB) of Bangabandhu Sheikh Mujib Medical University, Dhaka (BSMMU/2021/7555; date 21 August 2021). Informed written consent was taken from all patients and healthy controls after an adequate explanation of the purpose of the study.

## Results

### Inflammatory cytokine analysis

The serum levels of IP-10 among moderate, severe, and critically ill COVID-19 patients and healthy controls are shown in Table 1. In control, the mean IP-10 was 49.7 (± 79.3) pg/ml, whereas in the moderate group of patients, it was 96.0 (± 102.4) pg/ml, 613.8 (± 880.2) pg/ml in the severe group, and 1536.3 (± 1534.1) pg/ml in the critically ill group of patients. Statistical analysis shows that there is a significant statistical difference among the groups regarding the serum IP-10 level (*p* < 0.001).

**Table 1:** Association of serum level of IP-10 (pg/ml) among different categories of COVID-19 patients and healthy controls (*n* = 112)

Category of patients	IP-10 (pg/ml) (mean ± SD)	Mean rank	*p*-value
Control	49.7 ± 79.3	26.6	< 0.001
Moderate	96.0 ± 102.4	40.2	
Severe	613.8 ± 880.2	72.3	
Critical	1536.3 ± 1534.1	86.9	

The mean MCP-1 level was found to be 80.8 (± 65.1) pg/ml among healthy controls. In the moderate group of patients, it was 257.8 (± 378.9) pg/ml. In the severe group of patients, it was 496.1 ± 979.4 pg/ml, and lastly, in the critically ill group of patients, it was 1143.4 (± 1512.9) pg/ml. The statistical test revealed that there was a significant statistical difference among the groups regarding MCP-1 level (*p* < 0.001) (Table 2).

**Table 2:** Association of serum level of MCP-1 (pg/ml) among different categories of COVID-19 patients and healthy controls (*n* = 112)

Category of patients	MCP-1 (pg/ml) (mean ± SD)	Mean rank	*p*-value
Control	80.8 ± 65.1	30.6	< 0.001
Moderate	257.8 ± 378.9	48.8	
Severe	496.1 ± 979.4	66.9	
Critical	1143.4 ± 1512.9	79.7	

Table 3 shows the mean level of MIP-1α among the different comparison groups. Among healthy controls, the level of MIP-1α was found to be 2.0 (± 1.3) pg/ml: 37.6 (± 87.6) pg/ml among people suffering from a moderate disease, 28.3 (± 39.6) pg/ml among patients with a severe disease, and lastly 47.1 (± 67.9) pg/ml among patients with a critical disease. The significant statistical difference among the groups regarding MIP-1α was observed (*p* < 0.001). Table 4 shows the mean level of IL-6 was 3.1 (± 1.6) pg/ml among healthy controls; in the moderate group of patients, it was 83.4 (± 148.1) pg/ml; in the severe group of patients, it was 57.5 (± 70.6) pg/ml; and lastly, in the critical group of patients, it was 108.3 (± 139.4). Significant statistical variation was observed among COVID-19 disease severity groups in terms of IL-6 level (*p* < 0.001).

**Table 3:** Association of serum level of MIP-1α (pg/ml) among different categories of COVID-19 patients and healthy controls (*n* = 112)

Category of patients	MIP-1α (pg/ml) (mean ± SD)	Mean rank	*p*-value
Control	2.0 ± 1.3	16.0	< 0.001
Moderate	37.6 ± 87.6	70.0	
Severe	28.3 ± 39.6	67.2	
Critical	47.1 ± 67.9	72.7

**Table 4:** Association of serum level of IL-6 (pg/ml) among different categories of COVID-19 patients and healthy controls (*n* = 112)

Category of patients	IL-6 (pg/ml) (mean ± SD)	Mean rank	*p*-value
Control	3.1 ± 1.6	17.6	< 0.001
Moderate	83.4 ± 148.1	67.8	
Severe	57.5 ± 70.6	62.2	
Critical	108.3 ± 139.4	78.4	

### Genotype of rs12252 SNP (42 T/C) of the IFITM3 gene among COVID-19 patients and healthy controls

Among the healthy controls, 21 (75.0%) had TT and 7 (25.0%) had TC while no patient had CC genotype. In the moderate group, 22 (78.6%) had TT, and 6 (21.4%) had TC, while no patient had CC genotype. In the severe group, 6 (21.4%) had TT, 19 (67.9%) had TC, and 3 (10.7%) had CC genotype. However, in the critical group, only one patient had a TT genotype, whereas 13 (46.4%) had a TC genotype and the remaining half of the patients had genotype CC. The Fisher exact test showed that there was a significant statistical difference among the groups regarding the genotype of rs12252 SNP (42 T/C) of the *IFITM3* gene (*p* < 0.001) (Table 5).

**Table 5:** Distribution of study population by genotype of rs12252 SNP (42 T/C) of the *IFITM3* gene (*n* = 112)

rs12252 SNP (42 T/C) of the *IFITM3* gene	Control, *n* (%)	Moderate, *n* (%)	Severe, *n* (%)	Critically ill, *n* (%)	*p*-value
TT	21 (75.0)	22 (78.6)	6 (21.4)	1 (3.6)	< 0.001
TC	7 (25.0)	6 (21.4)	19 (67.9)	13 (46.4)	
CC	0 (0.0)	0 (0.0)	3 (10.7)	14 (50.0)	

Table 6 shows the comparison of the genotype of rs12252 SNP (42 T/C) of the *IFITM3* gene among COVID-19 patients and healthy controls. A statistical association was observed when compared between the severe and critically ill patient groups with that of healthy control group (*p* < 0.001). In addition, a significant correlation was found when compared between the moderate disease group with that of severe and critical cases.

**Table 6:** Comparison of rs12252 SNP (42 T/C) of the *IFITM3* gene with different category of study population (*n* = 112)

Study population	rs12252 SNP (42 T/C) of the *IFITM3* gene				
	TT, *n* (%)	TC and CC, *n* (%)	OR	CI	*p*-value
Moderate	22 (78.6)	6 (21.0)	1.22	0.352–4.234	0.752
Control	21 (75.0)	7 (25.0)			
Severe	6 (21.0)	22 (78.6)	0.091	0.026–0.315	< 0.001
Control	21 (75.0)	7 (25.0)			
Critical	1 (3.6)	27 (96.4)	0.012	0.001–0.108	< 0.001
Control	21 (75.0)	7 (25.0)			
Moderate	22 (78.6)	6 (21.0)	13.444	3.751–48.191	< 0.001
Severe	6 (21.0)	22 (78.6)			
Moderate	22 (78.6)	6 (21.0)	99.000	11.073–885.089	< 0.001
Critical	1 (3.6)	27 (96.4)			
Severe	6 (21.0)	22 (78.6)	7.364	0.824–65.833	0.101
Critical	1 (3.6)	27 (96.4)			

## Discussion

In SARS-CoV-2 infection, following a cytokine storm, several cytokines are secreted in great amount that ultimately results in a SIRS. Due to a damaged microvascular system and subsequent activation of the coagulation system, small vasculitis and extensive microthrombi formation take place [18]. Here, serum cytokines such as IP-10, MCP-1, MIP-1α, and IL-6 play a major role in determining COVID-19 disease severity [5, 19]. 

This study revealed that the serum level of IP-10 has a significant statistical association with different categories of COVID-19 patients. This finding is identical to the study by Chen et al., who reported that the serum level of IP-10 was markedly increased in patients with severe COVID-19 disease when compared to patients with a non-severe disease (*p* < 0.001) [4]. In addition, a study carried out by Zhao et al. revealed that the IP-10 was significantly increased among COVID-19 patients suffering from both mild and severe diseases, when compared with healthy controls. But after reaching a peak within 2 weeks, serum IP-10 level sharply fell back to normal level within 4 weeks in patients suffering from a mild disease. But in case of patients suffering from a severe disease, IP-10 level started to decrease after 2 weeks but took longer than 4 weeks to reach the normal level [20]. Hence, based on our findings and compared to that with relevant literature, IP-10 could be used as a potential biomarker for the discrimination between severe and mild COVID-19 disease [5, 21]. 

In this study, significant association was observed in terms of MCP-1 level among the moderate, severe, critically ill COVID-19 patient groups. Hussein found that MCP-1 level was significantly increased among severe COVID-19 patients when compared with mild patients [22]. Ozger et al. showed that there was a significant statistical difference (*p* = 0.015) between MCP-1 levels in non-survivor and survivors of COVID-19 disease. Higher serum level of MCP-1 among critically ill and non-survivor COVID-19 patients possibly results from an ongoing monocyte efflux from the circulation [23]. Pons et al. reported that high MCP-1 level is associated with lung injury and proposed as markers for disease prediction in hospitalized patients with respiratory failure and death [24]. Hence, serum level of MCP-1 might be used as a diagnostic as well as a prognostic marker for the assessment of COVID-19 outcome, as it correlates with COVID-19 disease severity and mortality.

In this study, the serum level of IL-6 and MIP-1α were found statistically significant (*p* < 0.001) among different COVID-19 patient groups. Among severe COVID-19 patients, the serum levels of IL-6 and MIP-1α are not increased proportionately compared to the moderate disease group, which may be due to the time point of the disease course at which samples were obtained from patients. In the early phase of infection, cytokine levels are found quite high than late or recovery phase of illness. Chen et al. found that IL-6 levels increased significantly in the critically ill group than in the moderate disease group and has a statistically significant association (*p* < 0.001) with disease severity [25]. Ling et al. showed that serum level of MIP-1α in severe and critically ill patients, when compared to moderate and mild patients, has a significant statistical difference (*p* = 0.004) [26]. On the contrary, Balnis et al. found that serum levels of IL-6 (*p* = 0.53) and MIP-1α (*p* = 0.42) had no significant difference between the survivor and non-survivor COVID-19 groups [27]. This finding indicates that serum levels of IL-6 and MIP-1α are adequate predictors of disease severity but may not be sufficient to be used as a tool for predicting mortality in COVID-19 disease.

In this study, genotype of rs12252 (42 T/C) SNP of the *IFITM3* gene showed that the TT genotype was more prevalent among moderate (78.6%) COVID-19 patients, whereas CC genotype was more common in critically ill (50%) patients. Comparison of rs12252 (42 T/C) SNP genotype among different categories of COVID-19 patients and healthy controls showed statistically significant differences (*p* < 0.001). It is postulated that TT genotype of rs12252 (42 T/C) SNP of the *IFITM3* gene causes restriction of SARS-CoV-2 virus fusion with endosomal membrane and prevents the subsequent entry of genomic material into the cytoplasm and further progression of COVID-19 disease, whereas CC genotype acts as a risk factor for developing a severe disease due to loss of antiviral function of *IFITM3* gene by SNP [19]. Zhang et al. found that TT genotype was common among mild (17.86%) and survivor COVID-19 patients and CC genotype in severe and non-survivor (66.7%) patients [17]. Findings from this study corroborates with previous findings and asserts the notion that CC genotype is responsible for COVID-19 disease severity.

There are several limitations to this study. First, the sample size was low compared to many other studies, largely due to budgetary constraints. Besides, samples were obtained, and cytokine levels were measured at a certain time point during patients’ hospital stay and might have impacted the study results.

## Conclusion

Serum levels of IP-10 and MCP-1 are elevated in critically ill COVID-19 patients, but MIP-1α and IL-6 are not increased proportionately. Genotyping of rs12252 (42 T/C) SNP of the IFITM3 gene showed that TT genotype was more prevalent among a moderate patient group where CC genotype was found to be more frequent in critically ill COVID-19 patients.

## Data Availability

The data that support the findings of this study are available on request from the corresponding author. The data are not publicly available due to privacy or ethical restrictions.
